# Supplementary material for: Developing and validating a clinical prediction model to predict epilepsy-related emergency department attendance, hospital admission, or death: A cohort study protocol
Source: PLoS One. 2025 Nov 10;20(11):e0328809. doi: 10.1371/journal.pone.0328809 (PMC12599926; doi:10.1371/journal.pone.0328809)
Supplement: Attachment — Submitted filename: 0) Response to Reviewers.docx [file pone.0328809.s001.docx]

# **Response to Reviewers**

**Journal requirements:**

When submitting your revision, we need you to address these additional requirements.

1. Please ensure that your manuscript meets PLOS ONE's style requirements, including those for file naming. The PLOS ONE style templates can be found at

<https://journals.plos.org/plosone/s/file?id=wjVg/PLOSOne_formatting_sample_main_body.pdf> and

<https://journals.plos.org/plosone/s/file?id=ba62/PLOSOne_formatting_sample_title_authors_affiliations.pdf>

**Author response:** We have now formatted the manuscript to the journal’s specifications.

2. Your ethics statement should only appear in the Methods section of your manuscript. If your ethics statement is written in any section besides the Methods, please move it to the Methods section and delete it from any other section. Please ensure that your ethics statement is included in your manuscript, as the ethics statement entered into the online submission form will not be published alongside your manuscript.

**Author response:** Our ethics statement is in the specified correct location in the manuscript.

**Reviewer comments:**

5. Is the manuscript presented in an intelligible fashion and written in standard English?

PLOS ONE does not copyedit accepted manuscripts, so the language in submitted articles must be clear, correct, and unambiguous. Any typographical or grammatical errors should be corrected at revision, so please note any specific errors here.

Reviewer #1: Yes

Reviewer #2: No

**Author response:** We thank the reviewers for these responses. We would like to respectfully point out that according to the journal's guidelines as stated above, reviewers should specify any typographical or grammatical errors if they select "no" to this question. Reviewer #2 has not noted any specific errors, so we are unable to address them in this revision. Reviewer #1 feels the manuscript presented in an intelligible fashion and written in standard English, which is reassuring.

**Reviewer #1:** The title is somewhat lengthy and complex, which might make it less accessible to a broader audience. Simplifying the language or shortening the title could make it more digestible.

**Author response:** This is helpful suggestion, thank you. We have now shortened and simplified the title to the following: “Developing and validating a clinical prediction model to predict epilepsy-related hospital admission or death: a cohort study protocol”

**Reviewer #1:** The keywords you've provided for the study are relevant, however, increase specificity, avoid redundancy and decreases the numbers up to 5 only.

**Author response:** Noted with thanks. We have now amended the keywords to the following five: “prognostic model, epilepsy health data, accident and emergency attendance, inpatient admission, mortality”

**Reviewer #1:** The abstract is quite lengthy for a standard abstract, which typically aims to succinctly communicate the study's essential elements. It delves into specific details, such as the number of patients in different databases, which might be more appropriate for the main text of the paper or the methods section.

**Author response:** These points are fair and well received. We have now removed the following from the abstract: “Data are held for 60 million patients in England on CPRD, 3.1m in Wales on SAIL, 2.6m in Cheshire and Merseyside on CIPHA, and 250m across 19 countries in TriNetX” - and replaced it with “Sample sizes of over 100,000 PWE are expected across these research platforms” – which is much simpler.

**Reviewer #1:** While the introduction is divided into sections, each section is heavily loaded with information, some of which delve into specific details that may distract from the primary focus of the introduction. Each section is clearly labeled (e.g., Clinical prediction models, Seizure-related emergency department or hospital admissions, Epilepsy-related deaths), which is helpful. However, the narrative could more explicitly connect these sections to show how they build on each other to justify the study's necessity and aims more coherently.

**Author response:** We have now re-ordered and re-written the whole introduction, which now flows much more seamlessly and reads as follows:

# **Introduction**

Hospital admissions and deaths from seizures are important outcomes to people with epilepsy (PWE). To inform patient counselling and clinical guidance about such outcomes, statistically robust and clinically meaningful clinical prediction models (CPMs) of who is at high (and low) risk of these outcomes can be developed and validated.

## **Seizure-related emergency department or hospital admissions**

As we previously demonstrated [1], PWE are at substantially increased risk of seizure-related emergency department (ED) or hospital admission [2]. Indeed, seizures are the most common neurological cause of ED or hospital admission in England [2], and such admissions can be predictors of subsequent epilepsy-related death [3, 4]. However, undergoing ED or hospital admission for epilepsy is often clinically unnecessary and typically leads to little benefit for management of the epilepsy because treatment decisions in epilepsy are complex and require specialist expertise, training and guidance [2]. The admitted PWE are usually seen by junior doctors and physicians without particular expertise in epilepsy and frequently discharged without specialist consultation or referral [1, 2, 5], representing missed opportunity for risk mitigation at a time when this may be crucial [1]. Such admissions could be avoided by early prediction of high-risk groups (through providing non-specialists such as general practitioners (GPs), paramedics, and physicians with tools for early prediction) because epilepsy is an ambulatory-care-sensitive condition [2, 6, 7]; meaning the high-risk groups are amenable to preventive targeting with scheduled specialist and community resources, diversion to alternative care pathways, optimised self-management, and accelerated medication reviews [8, 9].

## **Epilepsy-related deaths**

As we previously demonstrated [5, 10], PWE are at significantly increased risk of premature death. Some of those deaths may be entirely unrelated to their epilepsy. However, a substantial proportion are epilepsy-related [10, 11]. These are operationally defined as any death listing epilepsy as an underlying or contributory cause within death records [5, 12, 13], in line with national guidance [14]. Although sudden unexpected death in epilepsy (SUDEP) is a common epilepsy-related death, we have shown it is equally common for epilepsy-related death to occur through other mechanisms including aspiration pneumonia, cardiac arrest, antiseizure medication (ASM) poisoning, drowning, and alcohol dependence [5, 10]. Most epilepsy-related deaths occur in young adults [5, 10]. When we looked at epilepsy-related deaths as a group, nearly 80% were potentially avoidable [5]. Therefore, it is becoming increasingly pragmatic to investigate epilepsy-related deaths as a group rather than focusing on each cause individually [11, 12, 15].

## **Clinical prediction models (CPMs)**

CPMs estimate the risk of a future clinical outcome in an individual patient based on information retrieved from their routinely collected health data including age, sex, ethnicity, comorbidities, previous treatments, and other characteristics [16]. Therefore, CPMs help guide and standardise shared clinical decision-making and service delivery by drawing attention to high-risk individuals (needing earlier or more invasive treatments), and low-risk individuals (needing less invasive treatments with fewer side-effects) [17]. This helps personalise clinical management strategies. CPMs also help patients understand their own risks by quantifying them in a succinctly presented tool to aid patient-doctor discussions about risk [18]. For example, our CHA₂DS₂-VASc score [19, 20] is a simple 7-item CPM using everyday clinical information to accurately predict risk of stroke after developing atrial fibrillation (AF). This helps guide subsequent anticoagulation treatment decisions and patient discussions. We made CHA₂DS₂-VASc freely available online to any clinician in the world at [www.mdcalc.com](http://www.mdcalc.com). This combination of simplicity of use, accuracy, and wide availability has translated into substantial global impact and recommended use of this CPM within National Institute for Health and Care Excellence (NICE) guidelines [21].

## **CPMs for clinically meaningful outcomes for PWE**

An effective way to help avoid epilepsy-related deaths is to develop CPMs to identify high-risk groups and thereby aid clinicians in prioritising their care [4]. For example, in non-specialist settings, where such patients often first present [1, 2], high CPM scores could prompt re-discussion about seizure safety, signposting to online resources for support (e.g. [www.epilepsy.org.uk/living/safety](http://www.epilepsy.org.uk/living/safety)), and rapid-access epilepsy clinic referral (rather than reserving such clinics for first-fit patients alone). The potential impact of such approaches is well recognised. For example, the asthma deaths review demonstrated that many deaths were likely to be preventable simply through better proactive clinical management, such as arranging for patients to be seen promptly after a hospital admission, and through following clinical guidelines [22]. In specialist settings, high CPM scores could prompt referral for epilepsy surgery earlier than would have otherwise been considered, new discussion in a multidisciplinary team meeting, further medication reviews, or implementation of seizure alarms and nocturnal supervision.

There are currently no CPMs for ED or hospital admissions in PWE. Work is planned to develop a risk prediction tool to estimate the benefits a person with epilepsy would receive if conveyed to ED and risks if not [8]. Additionally, there are few CPMs of mortality in PWE. Although our recently developed Scottish Epilepsy Deaths Study score [4] predicted epilepsy-related deaths as a group, its reliance on hand-searched medical records limited sample sizes to 224 cases/controls, which widened confidence intervals, reduced precision, and meant only four predictors could be included. The SUDEP-7 and SUDEP-3 inventories focused on predicting SUDEP alone [23], and therefore missed the remaining epilepsy-related deaths as a group. They were also drawn from small sample sizes of 19 and 28 patients, respectively, limiting their reliability. Similarly, the Personalised Prediction Tool [24] focused on SUDEP alone and had only 287 cases and 986 controls.

None of the above CPMs have been validated in independent, geographically distinct, datasets (so-called external validation), meaning they cannot currently be used in clinical practice [16]. The SUDEP and Seizure Safety Checklist [25] is expert consensus guidance rather than a CPM. Developing a validated CPM for epilepsy-related deaths was highlighted as a key recommendation for epilepsy research in recent NICE guidelines [26].

## **Study aims and potential benefits to PWE**

In line with the recommendations of NICE [26], we aim to develop and externally validate a CPM to predict the joint risk of two outcomes occurring within the next year in PWE [27]: A) seizure-related ED or hospital admission; and B) epilepsy-related death. We hypothesise the CPM will be able to identify individuals at high or low risk of either or both outcomes. This will give clinicians seeing PWE a validated tool to predict either or both of these important outcomes. This has not been done before despite both outcomes being common, potentially avoidable, devastating for patients and their families, and closely interrelated [1-5, 9]. We will develop guidance for clinicians on proposed actions to take based on the overall risk score. When implemented, we expect this work to contribute to a reduction in the number of seizure-related ED or hospital admissions and epilepsy-related deaths.

**Reviewer #1:** Ethics and dissemination AND Data management plan should be in the method section.

**Author response:** Thanks. Ethics and dissemination was already located in the methods. We have now moved the data management plan to the methods section as well.

**Reviewer #1:** please re write the discussion section.

**Author response:** We appreciate the reviewer's feedback. To effectively revise the discussion section, we would need specific guidance on the issues that need to be addressed.

**Reviewer #2:** You need to improve English used. Moreover, you need to write more information about your work

**Author response:** We appreciate the reviewer's feedback. To improve the English, we need specific instances where the language is inadequate. Additionally, we require more detailed guidance on the additional information needed about our work to ensure we address the reviewer's concerns accurately.
