# Supplementary material for: Developing and validating a clinical prediction model to predict epilepsy-related emergency department attendance, hospital admission, or death: A cohort study protocol
Source: PLoS One. 2025 Nov 10;20(11):e0328809. doi: 10.1371/journal.pone.0328809 (PMC12599926; doi:10.1371/journal.pone.0328809)
Supplement: Attachment — Submitted filename: 0.1) Responses to reviewers.docx [file pone.0328809.s002.docx]

**REVIEWER 1.1**

Reviewer #1: 1-Expanding on the rationale behind selecting these specific datasets (CPRD, SAIL, CIPHA, TriNetX) and how they cover a representative population would enhance the justification of the study's scope.

**AUTHOR 1.1**

Thank you for this helpful suggestion. We have now included the following justification, on page 6:

“There are several reasons for planning to study CPRD, SAIL, CIPHA and TriNetX. First, it provides a contingency plan should we fail acquire one or more of the datasets, e.g. owing to cost. Second, it allows for internal and external validation within and between the geographically distinct populations represented by each of these datasets, allowing the final model generated to be more widely generalisable, facilitating future clinical implementation. In particular, each dataset’s coverage adds value to understanding the model’s performance across different healthcare systems and demographics. For example, CPRD is strengthened by its extensive primary care representation, SAIL by its linkage to socio-demographic data, CIPHA allows real-time analytics with re-identification at source, and TriNetX has a substantial sample size, offering flexibility in selecting a large number of predictors.”

**REVIEWER 1.2**
2- More explicit guidelines on how predictors will be chosen and defined would ensure clarity and reproducibility.

**AUTHOR 1.2**

Thanks. We have now expanded the model development and validation section on page 8 as follows:

*“A priori* clinical expertise and data-driven variable selection will refine the variable list for modelling [4]. A blinded variable selection strategy [4] will be implemented *a priori* to reduce the list of CPM predictors down to a clinically pragmatic list of seven variables (or less) [19, 20]. The primary driver of which variables are selected will be Delphi-method-driven consensus amongst the clinical research team and our Public Advisors (people affected by epilepsy) on which predictors are most important clinically and are readily accessible in the datasets (i.e. missing data patterns will be incorporated into the decision-making) [4, 37]. Consensus will be reached through a series of workshops between the clinical research team and Public Advisors. A Delphi consensus exercise has the potential to highlight predictors beyond the scope of existing literature [37]. The focus will not be on the strength of the predictor (e.g. using backwards selection). This is because it is better to select predictors based on a wider body of clinical knowledge than to try to depend on statistical significance of results which may be sensitive to random variation in the data points due to sampling variability, as detailed elsewhere [38]. We will consider penalisation methods (e.g., *lasso*), [39, 40] where appropriate.”

**REVIEWER 1.3**
3-Some sentences are overly long and complex. Breaking these down into simpler sentences would improve readability, especially for non-expert readers.

**AUTHOR 1.3**

We have now simplified the relevant sentences as follows:

Page 2:

Old version

This retrospective open cohort study develops and externally validates a clinical prediction model (CPM) to predict the joint risk of two important outcomes occurring within the next year in people with epilepsy (PWE): A) seizure-related emergency department or hospital admission; and B) epilepsy-related death.

New version

This retrospective open cohort study develops and externally validates a clinical prediction model (CPM) to predict the joint risk of two important outcomes occurring within the next year in people with epilepsy (PWE). These are: A) seizure-related emergency department or hospital admission; and B) epilepsy-related death.

Page 2:

Old version

Routinely collected, anonymised, electronic health data from Clinical Practice Research Datalink (CPRD), Secure Anonymised Information Linkage databank (SAIL), Combined Intelligence for Population Health Action (CIPHA), and TriNetX research platforms will be used to study PWE aged ≥16 years having outcomes A and/or B between 2010–2022.

New version

Routinely collected, anonymised, electronic health data from Clinical Practice Research Datalink (CPRD), Secure Anonymised Information Linkage databank (SAIL), Combined Intelligence for Population Health Action (CIPHA), and TriNetX research platforms will be used. We will study PWE aged ≥16 years having outcomes A and/or B between 2010–2024 within these datasets.

Page 8

Old version

The focus will not be on the strength of the predictor (e.g. using backwards selection) as it is better to select predictors based on a wider body of clinical knowledge than to try to depend on statistical significance of results which may be sensitive to random variation in the data points due to sampling variability, as detailed elsewhere [37].

New version

The focus will not be on the strength of the predictor (e.g. using backwards selection). This is because it is better to select predictors based on a wider body of clinical knowledge than to try to depend on statistical significance of results which may be sensitive to random variation in the data points due to sampling variability, as detailed elsewhere [38].

**REVIEWER 1.4**

4-Ensure consistency in terminology (e.g., "epilepsy-related death" vs. "epilepsy-related mortality") to avoid confusion.

**AUTHOR 1.4**

Thanks. We have changed to now consistently report epilepsy-related death rather than epilepsy-related mortality, throughout the text.

**REVIEWER 1.5**
5-There are minor grammatical issues (e.g., subject-verb agreement, redundant phrases) that should be corrected in revision.

**AUTHOR 1.5**

We have corrected minor grammatical issues which we have been able identify. For example, on page 11:

Old version

Whilst the study protocol is limited to developing and externally validating a stand-alone CPM only, this will be sufficient to allow external users to consider adopting and implementing the CPM into their local clinical care settings, as highlighted in the Prognosis Research Strategy (PROGRESS) 3 guidelines [16].

New version

Whilst the study protocol is limited to developing and externally validating a stand-alone CPM only, this will be sufficient to allow external users to adopt and implement the CPM into their local clinical care settings, as highlighted in the Prognosis Research Strategy (PROGRESS) 3 guidelines [16].
