# Supplementary material for: Developing and validating a clinical prediction model to predict epilepsy-related emergency department attendance, hospital admission, or death: A cohort study protocol
Source: PLoS One. 2025 Nov 10;20(11):e0328809. doi: 10.1371/journal.pone.0328809 (PMC12599926; doi:10.1371/journal.pone.0328809)
Supplement: Attachment — Submitted filename: 0.1)_Responses_to_reviewers_auresp_3.docx [file pone.0328809.s003.docx]

**Reviewer #2:** I don’t have any comment to authors

**Reviewer #3:** In this manuscript the authors developed and evaluated a clinical prediction model (CPM) useful to predict epilepsy-related hospital admission and epilepsy-related mortality. The topic of the present study could be of interest to the reader, since research on this topic could increase the knowledge about the tool to manage fatal outcomes in epilepsy sector. However, this study presents some limitations:

**Reviewer 3.1:**

Some sentences are too long and contain technical information

**Author 3.1:**

Thank you. We have carefully reviewed the manuscript and shortened or restructured all overly long sentences, often by splitting them into shorter, clearer components. We have also simplified technical phrasing where appropriate. These edits are tracked in the revised manuscript.

**Reviewer 3.2:**

All the abbreviations should be reported extensively the first time they are mentioned (e.g ‘AF’ for ‘Atrial Fibrillation or “COPD” for “Chronic obstructive pulmonary disease”)

**Author 3.2:**

We have reviewed the manuscript and ensured that all abbreviations are spelled out in full on first use. The revised text reflects these changes.

**Reviewer 3.3:**

Check in the text (including “abstract”) the terms “epilepsy-related death” and “epilepsy-related mortality” trying to make the whole text homogeneous;

**Author 3.3:**

Thank you. We have carefully reviewed the manuscript, including the abstract, and confirm that we consistently use the term “epilepsy-related death” throughout. The term “epilepsy-related mortality” does not appear in the text.

**Reviewer 3.4:**

The Delphi methods steps should be clarify (please define what do you mean with “a series of workshops”, maybe “focus groups” or “group of discussion” could be more appropriate).”

**Author 3.4:**

We have revised the text to clarify that the “series of workshops” refers to structured focus groups involving clinicians and public advisors, in line with Delphi methodology.

**Reviewer 3.5:**

Discussions needs to be revised in order to be more informative and less vague/superficial.

**Author 3.5:**

Thank you. We have reviewed the Discussion section and made minor changes to improve clarity and specificity, particularly regarding how the model may be implemented and updated over time. We hope this addresses the reviewer’s concern.

**Reviewer 3.6:**

Finally, the paper is not easy to read and a revision about the English language as well as the technical terms is necessary.

**Author 3.6:**

We have made substantial revisions to improve readability, including simplifying technical language and restructuring long sentences. We hope the manuscript now reads more clearly and is easier to follow.

**Reviewer #4:** The present study protocol aims to develop and validate a clinical prediction model to predict epilepsy-related hospital admission or death.

The revised version of the manuscript is clearly-written and the methods are well-detailed. I found this protocol very interesting to read and I think that are a lot of implications for clinical practice and research.

There are a few minor points/comments for the authors:

**Reviewer 4.1**

-Title: As the so-called outcome A includes ‘seizure-related emergency department’ together with ‘hospital admission’, I would suggest to include also the first in the title.

**Author 4.1**

Many thanks. Both terms have been added to the title.

**Reviewer 4.2**

-Abstract-Methods: ‘Candidate predictors will include demographic, lifestyle, clinical, and management’. I would add ‘variables’ at the end of the statement.

**Author 4.2**

Thanks. We have added ‘variables’ at the end of the statement.

**Reviewer 4.3**

-Abstract- Conclusions: I would suggest not to the start the section with ‘This is the largest study…’. Please, revise this and other similar statements along the manuscript.

**Author 4.3**

Thanks. We have revised these accordingly to “large study” and removed the superlatives.

**Reviewer 4.4**

-Lay summary- first row: ‘Some people…’ I would include the exact number, if possible. ‘Some’ is vague.

**Author 4.4**

Thank you. We agree that precise figures can be helpful; however, in this case, estimates vary depending on the population and data source. To avoid presenting potentially misleading figures, we have chosen to use the more general phrasing “people with epilepsy,” which accurately reflects the elevated risk without oversimplifying the data.

**Reviewer 4.5**
Fourth row: Again, please revise the statement: ‘Our study is the first to do this’.

**Author 4.5**

Thanks. We have removed this superlative.

**Reviewer 4.6**
Row 11: ‘Giving clinicians the tool […]’. I would replace the latter with ‘Providing clinicians with the tool […]’.

**Author 4.6**

Thanks. We have replaced the latter with ‘Providing clinicians with the tool’, as helpfully suggested.

**Reviewer 4.7**

-Introduction-Row 1: ‘[…] outcomes to people […]’. I would replace the latter with ‘[…] for people […]’.

**Author 4.7**

Thanks. We have replaced ‘to people’ with ‘for people’

**Reviewer 4.8**

‘Seizure-related emergency department or hospital admissions’: check for consistency of using the plural ‘admissions’ along the manuscript.

**Author 4.8**

Thanks. We have, for consistency, replaced these with the singular ‘admission’, where relevant, instead of ‘admissions’.

**Reviewer 4.9**
‘CPMs for clinically meaningful outcomes for PWE’- row 6: ‘For example, the asthma deaths review […]’. I would say ‘[…] an asthma death review […]’.

**Author 4.9**

Thanks. We have now used ‘an asthma death review’

**Reviewer 4.10**
-Methods-study design-first 10 rows: I would reshape these by listing datasets using bullet points for each dataset.

**Author 4.10**

Great idea. Thanks. We have now listed with bullet points.

**Reviewer 4.11**

Importantly, I would suggest to use the TRIPOD checklist and include in a relevant section of the protocol.

**Author 4.11**

We have used the checklist and added details to the methods.

**Reviewer 4.12**
-Discussion- row 1: ‘This is the largest study […]: please, revise this (see my comment above).

**Author 4.12**

Thanks. We have removed the superlatives.
